# Estimating and adjusting for ancestry admixture in statistical methods for relatedness inference, heritability estimation, and association testing

**DOI:** 10.1186/1753-6561-8-S1-S5

**Published:** 2014-06-17

**Authors:** Timothy Thornton, Matthew P Conomos, Serge Sverdlov, Elizabeth M Blue, Charles YK Cheung, Christopher G Glazner, Steven M Lewis, Ellen M Wijsman

**Affiliations:** 1 Department of Biostatistics, University of Washington, Seattle, WA 98195, USA; 2 Department of Statistics, University of Washington, 313 Padelford Hall, Seattle, WA 98195, USA; 3 Department of Medicine, Division of Medical Genetics, University of Washington, Health Sciences Building, K-253, Box 357720, Seattle, WA 98195, USA

## Abstract

It is well known that genetic association studies are not robust to population stratification. Two widely used approaches for the detection and correction of population structure are principal component analysis and model-based estimation of ancestry. These methods have been shown to give reliable inference on population structure in unrelated samples. We evaluated these two approaches in Mexican American pedigrees provided by the Genetic Analysis Workshop 18. We also estimated identity-by-descent sharing probabilities and kinship coefficients, with adjustment for ancestry admixture, to confirm documented pedigree relationships as well as to identify cryptic relatedness in the sample. We also estimated the heritability of the first simulated replicate of diastolic blood pressure (DBP). Finally, we performed an association analysis with simulated DBP, comparing the performance of an association method that corrects for population structure but does not account for relatedness to a method that adjusts for both population and pedigree structure. Analyses with simulated DBP were performed with knowledge of the underlying trait model.

## Background

Principal component analysis (PCA) and model-based estimation of ancestry are two widely used approaches for the detection and correction of population structure in admixed populations. The top principal components (PCs) from PCA can be used as covariates in a generalized linear model to protect against confounding resulting from population stratification in genetic association studies [1]. Individual ancestry estimates from widely used software programs, such as STRUCTURE [2], FRAPPE [3], and ADMIXTURE [4], can also be used for population stratification inference and correction. PCA and individual ancestry estimation methods have been shown to give reliable inference for ancestry in admixed samples with unrelated individuals. We evaluate the performance of PCA and the model-based individual ancestry estimation method ADMIXTURE in Mexican American pedigrees provided by the Genetic Analysis Workshop 18 (GAW18). We also estimate the heritability of the first simulated replicate of diastolic blood pressure based (DBP), and we compare heritability estimates calculated using pedigree-based kinship coefficients versus empirical kinship coefficients that are estimated from single-nucleotide polymorphism (SNP) genotype data. We also perform an association analysis with SNP genotype data and the first simulated replicate of DBP. Then we compare the EMMAX association method [5], which is a linear mixed-model approach that accounts for pedigree and population structure, with an association analysis using the PLINK software [6], where the top 10 PCs from a PCA are included as covariates in a linear regression analysis to account for population structure.

## Methods

### Individual ancestry estimation

We performed both supervised and unsupervised structure analyses for the GAW18 sample using the ADMIXTURE software. For the supervised structured analysis, proportional European, African, Native American, and East Asian ancestry was estimated using SNP genotype data for the odd-numbered chromosomes. There are 955 individuals in GAW18 who have available genotype data. We set the number of ancestral populations to 4 in the ADMIXTURE analysis, where the CEU and YRI samples of release 3 of phase III of the International Haplotype Map Project (HapMap) [7] were used as surrogates for European and African ancestry, respectively, and the Human Genome Diversity Project (HGDP) [8] samples from the Americas and East Asia were used for Native American and East Asian ancestry. The surrogate HGDP sample for Native American ancestry includes 8 Surui, 22 Maya, 13 Karitiana, 14 Pima, and 6 Colombian individuals. We used 242,566 autosomal SNPs that were genotyped in all three data sets (HapMap, HGDP, and GAW18) for the supervised analysis. The unsupervised analysis with ADMIXTURE was similar to the supervised analysis except that the reference HapMap and HGDP samples were not included in the ancestry analysis.

### Principal component analysis with pedigrees

PCA has been shown to account for population stratification in samples with unrelated individuals. In samples with related individuals, however, the top PCs from standard PCA (sPCA) may not adequately account for population structure because of the complicated covariance structure of the genotypes among relatives. For genetic studies that contain nuclear families, Zhu *et al *[9] proposed a method for obtaining ancestry-informative PCs by first performing sPCA on the genotyped parents in the pedigrees and then using SNP weights from the PCA on the parents to obtain PCs for offspring. We extended the Zhu *et al *approach to general pedigrees by (a) selecting a set of genetically unrelated individuals and performing PCA on this unrelated set and (b) using the SNP weights from the unrelated set to obtain PCs for all remaining individuals in the sample. We name this approach R-PCA, where *R *indicates that this PCA method accounts for relatedness in the sample. We apply both sPCA and R-PCA to the GAW18 sample using 100,000 SNPs that were selected at random, without replacement, from the set of genotyped SNPs on the odd-numbered chromosomes.

### Relatedness estimation in GAW18

Kinship coefficients and identity-by-descent (IBD) sharing probabilities were estimated for all pairs of genotyped individuals in the GAW18 sample with the REAP (relatedness estimation in admixed populations) method [10]. REAP gives robust IBD sharing probability and kinship coefficient estimates in admixed populations by using individual-specific allele frequencies that are calculated by conditioning on estimated genome-wide ancestry. The REAP relatedness estimates were calculated using the ADMIXTURE-estimated individual ancestry proportions and ancestral population allele frequencies from the supervised ancestry analysis, and with the 242,566 overlapping SNPs in the HapMap, HGDP, and GAW18 samples (discussed earlier).

### Association testing with simulated diastolic blood pressure phenotype

We performed an association analysis with the first simulated replicate of DBP and SNPs that were genotyped on the odd-numbered chromosomes. We used the first time point for DBP and adjusted the DBP phenotype for age, sex, and current use of antihypertensive medications. The PLINK software was used to perform a linear regression association analysis, where the top 10 PCs, calculated from the EIGENSOFT software [1], were used as covariates in the linear regression model to adjust for population structure in the sample. Note that the association analysis conducted with PLINK corrects for population structure but does not account for relatedness in the sample. We also performed an association analysis using the EMMAX method. EMMAX is a linear mixed-model approach that uses an empirical covariance matrix, estimated using genome-screen data, to account for both pedigree and population structure.

### Heritability estimation of simulated diastolic blood pressure phenotype

There were 845 individuals in GAW18 for whom both SNP genotype data and phenotype information were available for simulated DBP, and we computed estimates of heritability using these sample individuals. Heritability estimates were calculated using a pedigree-based kinship coefficient matrix, an empirical matrix with REAP-estimated kinship coefficients, and a hybrid matrix of the two. The hybrid matrix uses REAP estimated kinship coefficients only for pairs of individuals who are related according to the available pedigree information. Individuals who are not related based on the pedigrees have a kinship coefficient value of 0 in the hybrid matrix, which corresponds to the same kinship coefficient value for the unrelated pairs in the pedigree-based kinship coefficient matrix. We implemented a restricted maximum likelihood estimation procedure to compute the variance components for heritability estimation.

## Results

### Individual ancestry estimation

**Figure 1:**
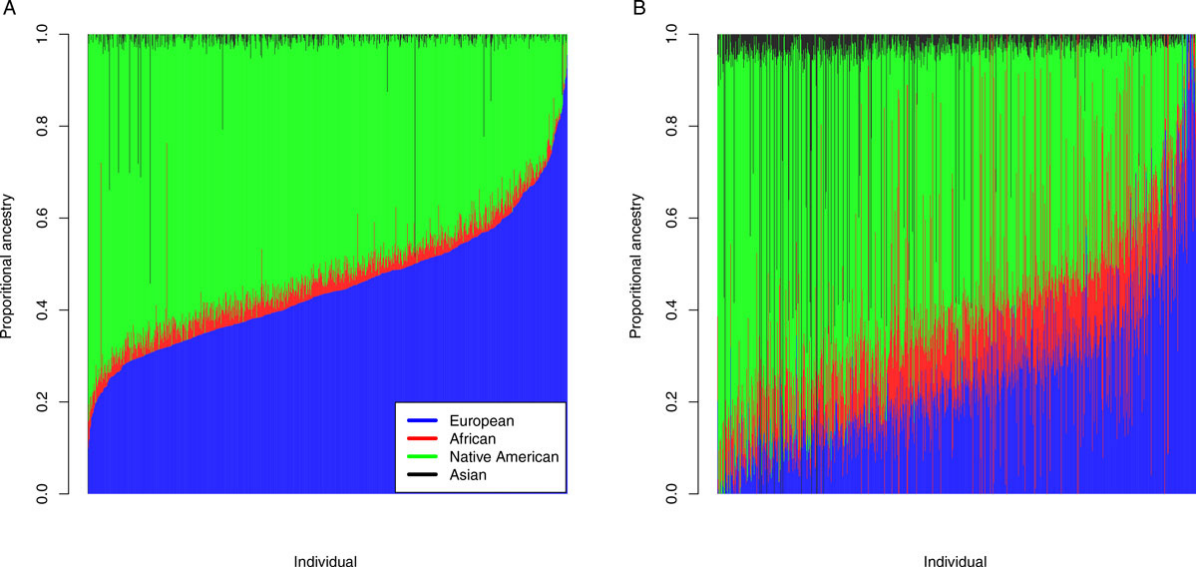
**Individual ancestry analysis**. Bar plots of individual-ancestry estimates from a supervised and an unsupervised structure analysis, respectively, with the ADMIXTURE software program for 955 genotyped Genetic Analysis Workshop 18 (GAW18) individuals. A) GAW18-supervised individual ancestry analysis. B) GAW18-unsupervised individual ancestry analysis. Each individual is represented by a vertical bar in A and B. The 4 ancestral populations in B that are inferred by ADMIXTURE in the unsupervised analysis are represented by the colors blue, red, green, and black. The order of the individuals is the same for A and B.

Figure 1 presents bar plots of individual ancestry estimates for the supervised and unsupervised ancestry analysis from the ADMIXTURE software. Figure 1A shows the results for the supervised ADMIXTURE analysis in which the HapMap CEU and YRI samples and the HGDP Native American and East Asian samples were included in the analysis as fixed groups; proportional ancestry was estimated for the 955 GAW18 individuals with available SNP genotype data. In the bar plot of the supervised ADMIXTURE ancestry estimates (Figure 1A), individuals are represented by vertical bars, and the GAW18 individuals in this figure are arranged in increasing order (left to right) of genome-wide European ancestry proportion. On average, there is modest African and East Asian ancestry in the GAW18 sample, where the average proportional African ancestry is 3.6% with an SD of 2.9%, and the average proportional East Asian ancestry is 1.2% with an SD of 4.8%. Three individuals have high proportional East Asian ancestry, where one individual is 100% East Asian and two admixed individuals have ADMIXTURE-estimated East Asian ancestry proportions of 48.5% and 54.3%. Two individuals have high African ancestry, with estimated proportional ancestry of 44.5% and 50.4% from Africa. Most of the GAW18 ancestry is European and Native American. Proportional European and Native American ancestry is quite variable; proportional European ancestry ranges from 0% to 95.5%, with a mean and SD of 44.9% and 14.0%, respectively, and proportional Native American ancestry ranges from 0% to 83.9%, with a mean and SD of 50.3% and 13.9%, respectively.

Figure 1B presents a bar plot of the results from the unsupervised ADMIXTURE analysis, where the order of the GAW18 individuals is the same as in Figure 1A. Comparing Figures 1A and 1B, we see that the unsupervised individual ancestry proportions are much more variable than the supervised analysis. One of the ancestry components in the unsupervised analysis reflects pedigree membership in family 5. A number of individuals in this family have estimated proportional ancestry that is greater than 99% for this component and 64 of the 68 genotyped individuals in this family have the highest estimated proportions for this component. Another ancestry component is heavily influenced by families 4 and 6 in the unsupervised analysis, where the highest 114 proportions for this component are from these two families.

The individual ancestry results with ADMIXTURE illustrate that (a) an unsupervised analysis may not give appropriate proportional ancestry estimates in samples with related individuals and (b) a supervised ancestry analysis can give reliable individual ancestry estimates in related samples with ancestry admixture, as was previously demonstrated in an analysis of admixed population samples with relatedness [10].

### Principal components analysis with pedigrees

**Figure 2:**
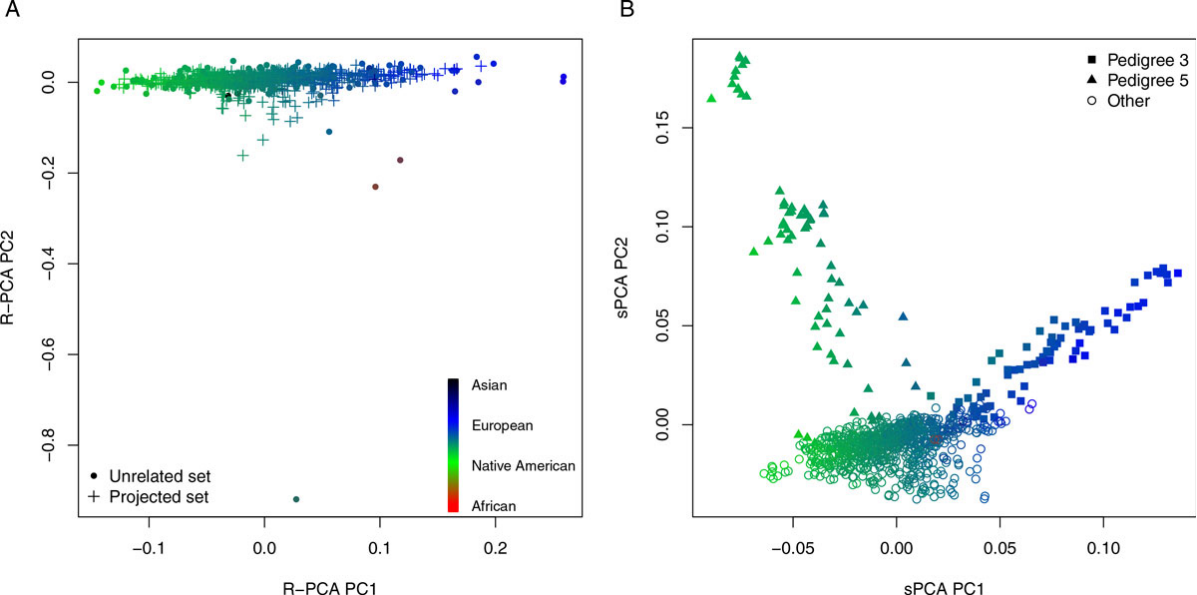
**Principal components analysis**. The top two principal components from R-PCA (A) and sPCA (B) are plotted against each other. The color of each point in the figures corresponds to an individual's ADMIXTURE-estimated ancestry.

We find that the top PC from R-PCA is highly correlated with the Native American ancestry component from the supervised ADMIXTURE analysis discussed in the previous subsection; the linear regression model for predicting Native American ancestry from the top PC has an *r*^2 ^= 0.92 (i.e., 92% of the ADMIXTURE-estimated proportional Native American ancestry is explained by the top PC from R-PCA). The top PC for sPCA, however, does not capture Native American ancestry as well as R-PCA, with an *r*^2 ^value of 0.82. Using the top 10 PCs from sPCA as predictors in a linear regression model with Native American ancestry as the response yields an *r*^2 ^= 0.86. The top 32 PCs from sPCA are needed as predictors in the regression model to match the proportional variance explained for Native American ancestry by the first PC from R-PCA. Proportional European ancestry explained by the top PC from R-PCA and PCs from sPCA are similar to the results for the Native American ancestry component because these two ancestry components are almost perfectly negatively correlated. The top 10 PCs from R-PCA have *r^2 ^*values of 0.74 and 0.69 for explaining proportional East Asian and African ancestry, respectively. In contrast, the top 10 PCs from sPCA have *r*^2 ^values of 0.012 and 0.097, respectively, for East Asian and African proportional ancestry. Figure 2 displays scatter plots of the top 2 PCs from R-PCA and sPCA. Figure 2B illustrates that the top 2 PCs from sPCA largely reflect family structure, where PC 1 is heavily influenced by family 3, and family 5 drives PC 2. In contrast, Figure 2A illustrates that the top two PCs from R-PCA reflect ancestry and not pedigree structure.

### Relatedness estimation in GAW18

The vast majority of the REAP estimated kinship coefficients are consistent with the known pedigree relationships. There are, however, some cryptically related individuals from different families who were identified with REAP. Table 1 gives 14 REAP-inferred relative pairs linking families 5 and 6, families 7 and 10, and families 21 and 25.

**Table 1:** REAP-inferred close relative pairs from different families

Individual 1		Individual 2			
			
Family number	Identification number	Family number	Identification number	REAP kinship coefficient	REAP IBD = 0 probability
5	T2DG0500371	6	T2DG0600393	0.05	0.76
5	T2DG0500380	6	T2DG0600393	0.03	0.85
5	T2DG0500389	6	T2DG0600393	0.04	0.82
7	T2DG0701151	10	T2DG1000614	0.05	0.82
7	T2DG0701151	10	T2DG1000615	0.04	0.87
7	T2DG0701151	10	T2DG1000616	0.04	0.82
7	T2DG0701151	10	T2DG1000639	0.03	0.86
21	2DG2100948	25	T2DG2501033	0.05	0.76
21	T2DG2100951	25	T2DG2501033	0.05	0.78
21	T2DG2100961	25	T2DG2501033	0.04	0.84
21	T2DG2100962	25	T2DG2501033	0.03	0.86
21	T2DG2100978	25	T2DG2501033	0.03	0.87
21	T2DG2100972	25	T2DG2501033	0.03	0.85
21	T2DG2100973	25	T2DG2501033	0.03	0.84

The following abbreviations are used: *IBD*, identity by descent; *REAP*, relatedness estimation in admixed populations.

### Association testing with simulated diastolic blood pressure phenotype

**Figure 3:**
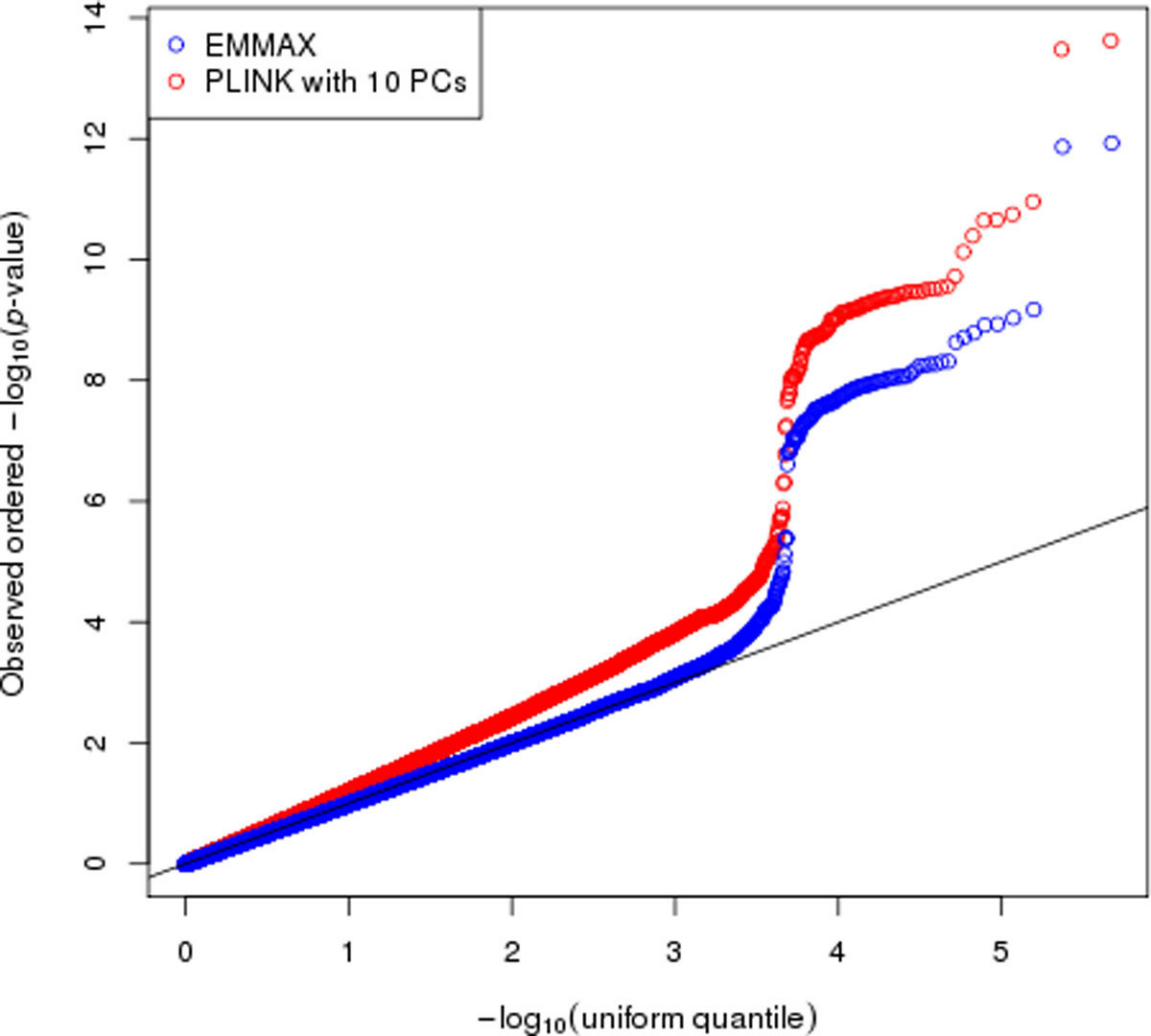
**Quantile-quantile (Q-Q) plot for EMMAX and PLINK association analysis with simulated diastolic blood pressure (DBP)**. Q-Q plots of ***p***-values from EMMAX and PLINK with the top 10 PCs included as covariates for the first simulated replicate of DBP, plotted on the -log_10 _scale. Red and blue circles in the figure correspond to the association results for PLINK and EMMAX, respectively.

**Figure 4:**
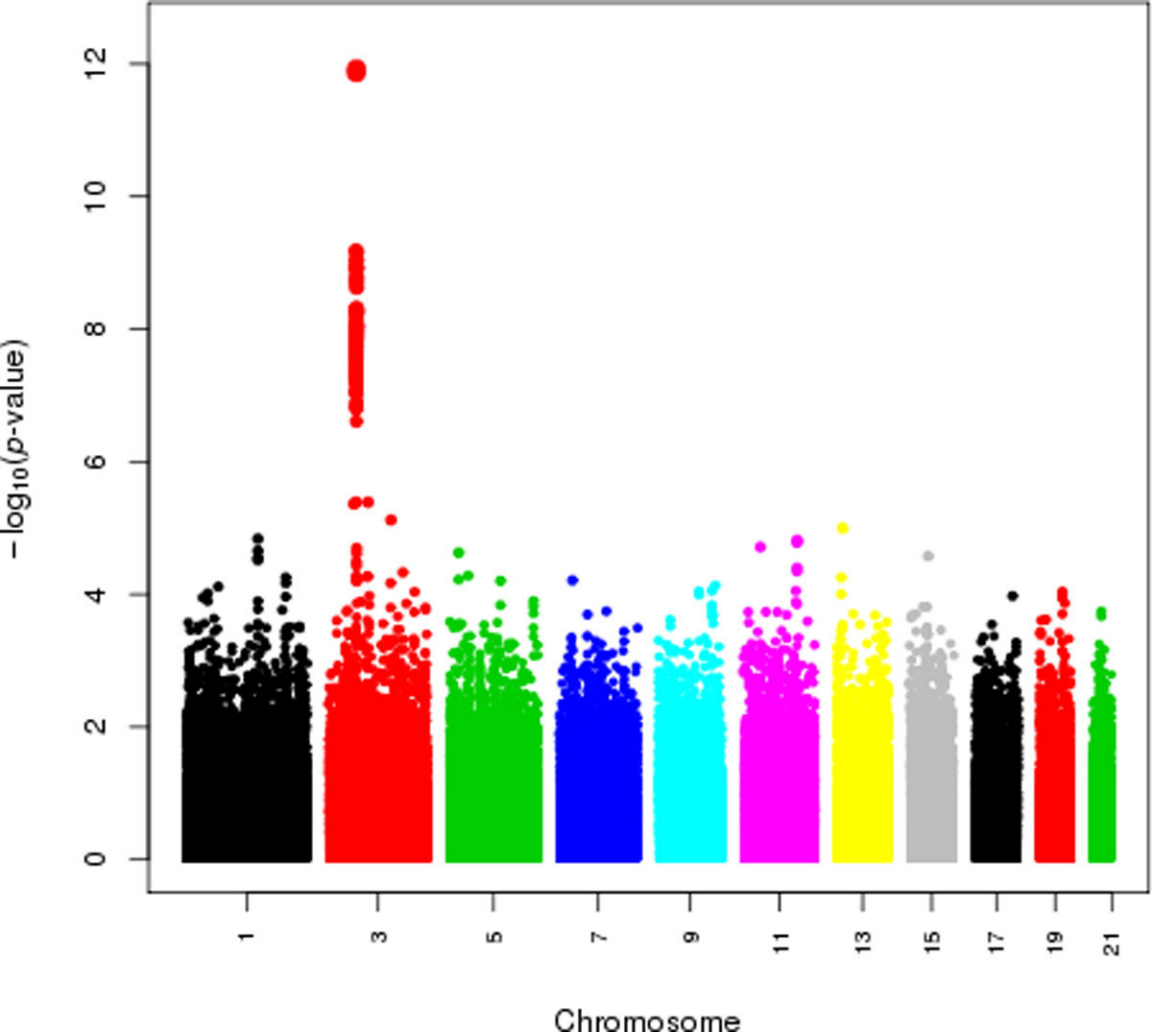
**Association results for EMMAX with simulated diastolic blood pressure (DBP)**. The Manhattan plot of ***p***-values from EMMAX for the first simulated replicate of DBP is plotted on the -log**_10 _**scale. The x- and y-axes show chromosome number and -log**_10 _**(***p***-value), respectively.

Figure 3 displays quantile-quantile (Q-Q) plots for the PLINK and EMMAX association analyses of the first simulated replicate of DBP. From this figure, one can see that PLINK is not properly calibrated, and the *p*-values are systematically inflated for the expected null *p*-values that are less than 0.01. The genomic control inflation factor [11], λ, for the PLINK association analysis is 1.3. In contrast, λ = 0.97 with EMMAX, indicating that the method is slightly conservative for the association analysis of the first simulated replicate of DBP. Most of the EMMAX *p*-values fall directly on or very close to the 45-degree line in the Q-Q plot until approximately *p *= 0.001. The large increase in the -log(*p*-values) after this point corresponds to functional variants for the simulated DBP phenotype. Figure 4 is a Manhattan plot of the EMMAX *p*-values for SNPs on the odd-numbered chromosomes. EMMAX identifies a number of highly significant associations with simulated DBP and SNPs in the functional region of the MAP4 gene on chromosome 3, where rs11711953, rs11706549, and rs11716779 are the three most significant SNPs with corresponding *p*-values of 1.2e-12, 1.4e-12, and 6.7e-10, respectively.

### Heritability estimation of simulated diastolic blood pressure phenotype

Heritability estimates of the first simulated replicate of DPB are remarkably close when using a pedigree-based kinship coefficient matrix, a REAP-estimated kinship coefficient matrix, and a hybrid of the two in the variance components analysis, where the corresponding heritability estimates (and 95% confidence interval) are 34.3% (21.7%-54.2%), 30.2% (18.3%-50.0%), and 33.1%(20.5%-53.4%), respectively. Estimation of heritability from variance components of pedigrees can lead to inflated heritability estimates resulting from confounding of shared environment of relatives. Recent work has been proposed to address this issue in GWAS samples by excluding relatives from a large-scale population study with empirical kinship coefficients that are greater than a specified threshold [12].

## Conclusions

Supervised individual ancestry analyses can give reliable proportional ancestry estimates in admixed population samples, provided that the surrogates for the ancestral populations are well represented in the analysis, but unsupervised individual ancestry methods perform poorly in this setting. We demonstrated that the top PCs in sPCA may not adequately reflect ancestry in samples with pedigrees from admixed populations. In contrast, our proposed method, R-PCA, can be used to obtain ancestry informative PCs in samples containing general pedigrees with known relatedness. Using the REAP relatedness estimation method, we obtained empirical kinship coefficients and IBD sharing probabilities in the GAW18 sample; we confirmed pedigree relationships and identified cryptic relationships that have not been reported. We also performed association testing with the first simulated replicate of DBP using the EMMAX association method, and genome-wide significant associations were detected with functional variants in the MAP4 gene on chromosome 3. Finally, we estimated the heritability of the first simulated replicate for DBP in the GAW18 pedigrees, and we found that the heritability estimates were remarkably similar when computing variance components with an empirical kinship coefficient matrix calculated with the REAP method, a pedigree-based kinship coefficient matrix, and a hybrid of the two kinship matrices.

## Competing interests

The authors declare that they have no competing interests.

## Authors' contributions

All authors participated in study design and analysis. TT drafted the manuscript, and all authors edited and approved the final manuscript.
